# Effect of acupuncture on pain and substance P levels in middle-aged women with chronic neck pain

**DOI:** 10.3389/fneur.2023.1267952

**Published:** 2023-10-19

**Authors:** Jade Heejae Ko, Seung-Nam Kim

**Affiliations:** College of Korean Medicine, Dongguk University, Seoul, Republic of Korea

**Keywords:** acupuncture, chronic neck pain, substance P, middle-aged women, neck disability index

## Abstract

Chronic neck pain is a leading health issue affecting a significant proportion of the global population. Multiple treatment options for chronic neck pain include anti-inflammatory drugs and analgesics. Acupuncture has been widely used for the treatment of chronic pain. In this study, we aimed to determine the efficacy of acupuncture for female patients with chronic neck pain. Twenty-three participants were enrolled in the study, and participants waited 4 weeks without acupuncture treatment and then received 4 weeks of treatment. One-way ANOVA with repeated measures was used to determine differences in the visual analogue scale (VAS), neck disability index (NDI), and substance P (SP) over time. The subjects’ pain intensity and degree of disability due to neck pain were measured as primary outcomes. SP in the blood was also analyzed as a secondary outcome. There was no significant difference between the VAS score and NDI value of baseline and after 4 weeks waiting. However, there was an improvement in both VAS and NDI after 4 weeks treatment. SP level was decreased after 4 weeks treatment. We could conclude that acupuncture is effective in alleviating chronic neck pain. Moreover, our findings revealed the efficacy of acupuncture on chronic pain with potential underlying biological mechanisms.

## 1. Introduction

Neck pain is a highly prevalent musculoskeletal disorder affecting individuals, families and healthcare systems of countries with substantial economic burdens. The Global Burden of Disease Study between 1990 and 2017 studied trends and prevalence of neck pain, and neck pain was reported as a serious public health problem, still affecting a significant proportion of the global population (1, 2). Popular analgesics such as non-steroidal anti-inflammatory drugs, acetaminophen, and cyclooxygenase 2 inhibitors relieve pain by suppressing the inflammatory process and have been commonly used as pharmacological interventions for musculoskeletal diseases, including neck pain (3, 4).

There are also non-pharmacological treatments such as physiotherapy, manual treatment, and massage therapy (5–7). Acupuncture has been gaining increasing interest for its favorable clinical outcomes in various chronic pains (8–10). Studies have shown a significant improvement in the visual analogue scale (VAS) score in subjects with acute or chronic musculoskeletal diseases such as low back pain after receiving acupuncture treatments (11–13). Nevertheless, there are still controversies over the clinical mechanisms by which insertion and stimulation by needles alleviate chronic pain.

Substance P (SP) is a mediator involved in various physiological processes, such as neuroinflammation and pain transmission (14, 15). It is broadly distributed in both the central and peripheral nervous system, and some studies have reported control of diseases, such as inflammatory muscle pain, cancer, and colitis, by mediating SP levels in animal models (16, 17). It has been hypothesized that neurological stimulation by needles at acupoints would cause physiological changes, mediating SP in serum (18).

The effect of acupuncture on chronic neck pain and its underlying mechanism is not well explored. In this study, we aimed to investigate the effect of acupuncture on chronic neck pain with different measurements and to further examine the potential biological connection between pain and SP level.

## 2. Methods

### 2.1. Study participants

Participant recruitment was performed through advertisement using local flyers in Goyang City, Gyeonggi-do and Seoul City, South Korea, from April 2018 to February 2019. A pre-screening survey was conducted on volunteered participants by assessing numeric pain scale (0–10 points), current medication, and questionnaires based on inclusion and exclusion criteria. Eligible participants were women aged between 40 to 60 years having neck pain for more than 12 weeks (VAS score ≥30.0 mm) (19). The inclusion criteria for participation were the absence of radiating pain and medication or other pain-related treatments. The exclusion criteria were pregnancy, spondylolisthesis, spondylitis, and infectious diseases, potentially affecting the treatment outcome.

### 2.2. Ethical approval and consent

Ethical approval for this study was obtained from the Institutional Review Board of Kyonggi University (KGU-20171222-HR-026). This study was registered with the Korean Clinical Trial Registry and WHO Clinical Trial Registry (KCT0005363, registered April 3rd, 2018, https://cris.nih.go.kr/cris/en/). The study procedures and potential risks were explained to each participant, and written informed consent was obtained prior to the study enrollment.

### 2.3. Study procedures

**Figure 1:**
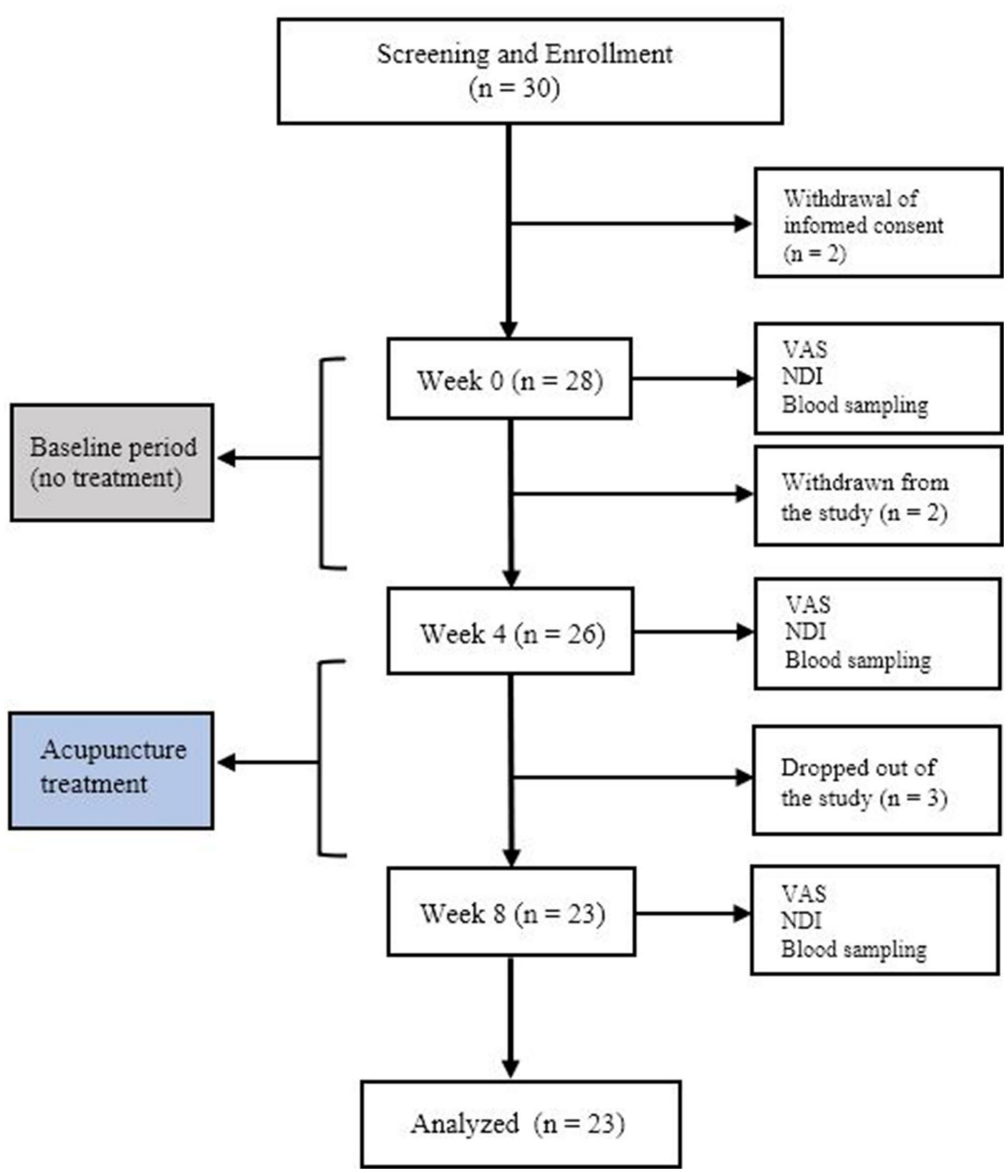
Flow chart of the current study.

We primarily screened 29 participants to be enrolled in the study. After the first blood sampling (20), two participants dropped out due to perceived discomfort in blood sampling. Four participants withdrew from the study due to not being able to comply with the treatment schedule. Twenty-three subjects were ultimately included in the study and the further outcome analysis (Figure 1). All the outcome measures were assessed by an independent investigator. The investigator was also blinded to the treatment procedure and each patient’s treatment.

### 2.4. Acupuncture protocol

**Figure 2:**
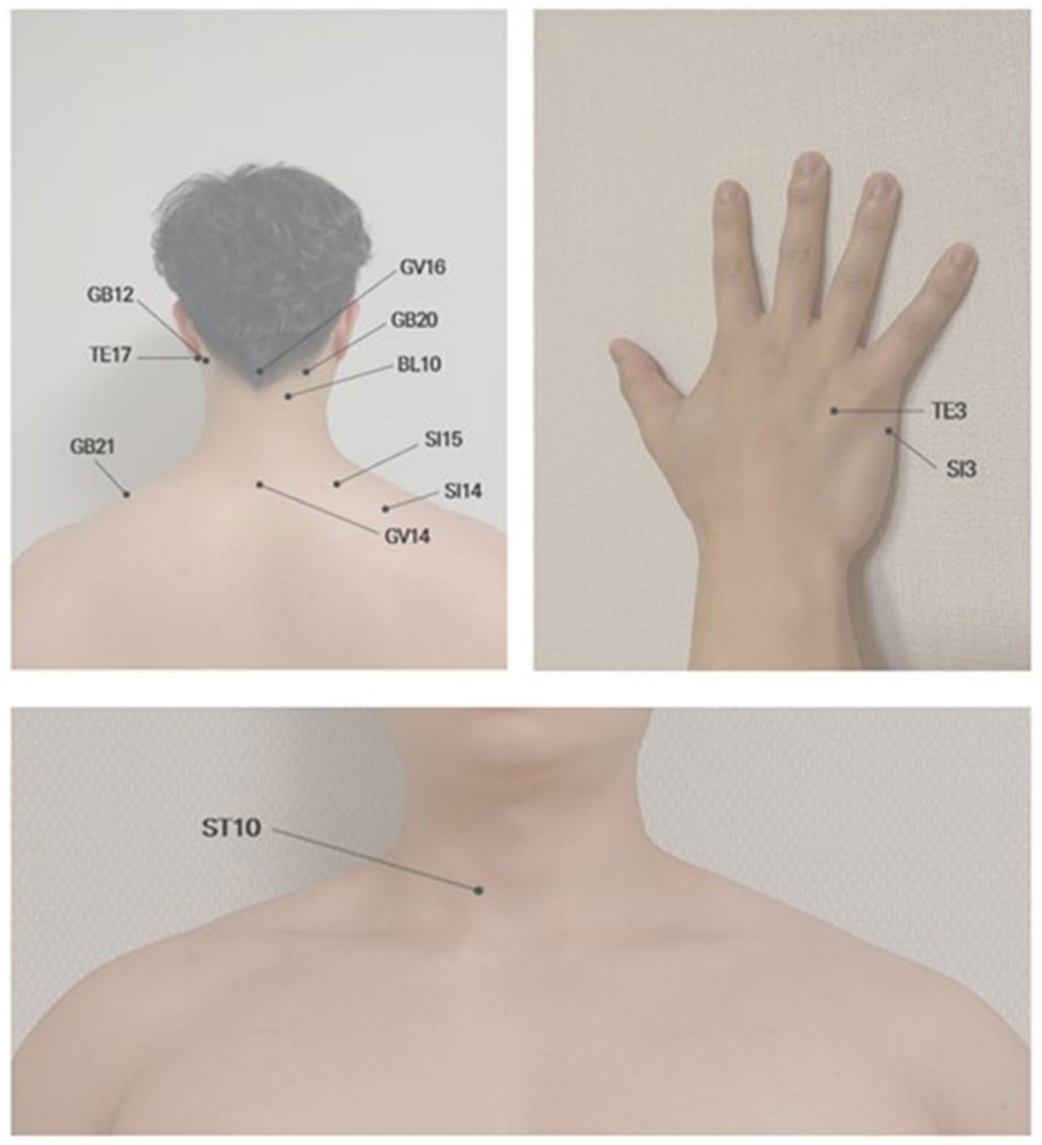
Location of the 2 distal acupoints and 10 proximal acupoints used in the current study.

All the subjects received acupuncture treatment at two proximal acupoints (GV14 and GV16) and ten acupoints (BL10, GB20, GB12, GB21, TE3, TE17, ST10, SI3, SI14, and SI15), which are bilaterally symmetric. Acupoints were located near the head, cervical vertebrae, neck and shoulder (Figure 2). The practitioner used disposable sterile needles (0.25 × 0.30 mm) and inserted the needles to a depth of 10–20 mm using a guide tube. The needles remained in the acupoints for 15 min, and each treatment session took approximately 20 min, with relaxation time before and after each treatment. A licensed oriental medicine doctor performed all the acupuncture treatments.

### 2.5. Primary outcome measures

#### 2.5.1. Visual analogue scale

Neck pain intensity was evaluated using VAS. Patients marked the point on a 10 cm horizontal line that best estimated their intensity of pain. The left end (0.0 cm) represented “no pain,” and the right end (10.0 cm) represented “extreme pain” (21). The measurements from the left end of the scale to the patients’ marks were recorded in centimeters and analyzed as their pain intensity.

#### 2.5.2. Neck disability index

Neck pain-related disability was assessed by the Korean version of NDI to determine patients’ perceived pain levels and degree of interference with daily activities (22). The NDI questionnaire covers ten items: pain intensity, personal care, lifting, reading, headaches, concentration, work, driving, sleeping, and recreation. Each item is scored on a 0 (no disability) to 5 (full disability) scale with a maximum total score of 50 points or 100% (23). If a subject responded that they do not drive, we recorded the NDI out of 45 and converted the score to a percentage.

The VAS and NDI measures were completed by each subject before the 4 weeks waiting (baseline), after the 4 weeks waiting (week 4), and after the 4 weeks treatment (week 8).

### 2.6. Secondary outcome measures

Blood sampling was conducted at three different time points (baseline, week 4, and week 8). The blood from each subject was collected into anticoagulant (EDTA)-treated tubes and prepared by centrifuging the whole blood at 3000 RPM for 15 min at 4°C. Plasma was then transferred into a new microtube and stored at −80°C until analysis. Serum SP levels were measured by enzyme-linked immunosorbent assay (ELISA) (KGE007, R&D, United States) and multimode plate reader (VICTOR Nivo, PerkinElmer, United States).

### 2.7. Statistical analysis

All data were analyzed using SPSS version 24.0 (IBM SPSS Statistics, Armonk, NY, United States). Statistical significance was set at *p* < 0.05 for all analyses. An independent *t*-test or chi-square test was performed to compare all variables at baseline between groups. One-way ANOVA with repeated measures at three different time points as main factors were used to test whether pain level significantly differed from baseline in VAS, NDI, and serum SP levels. When a group-by-time interaction was significant, the significance of the difference from baseline was tested by paired *t*-test in each group. The Greenhouse–Geisser correction was applied upon violating the assumption of Mauchly’s test of sphericity.

## 3. Results

### 3.1. Baseline characteristics

Baseline characteristics of the subjects, including age, body mass index (BMI), current medication, and duration of neck pain, are displayed in Table 1. The average age of the subjects (*n* = 23) was 54.17, and the average BMI was 24.28. Duration of neck pain was less than 3 years for all the subjects, with a mean of 2.65 years. Eighteen subjects (78.3%) were not on any medications, and the types of medication that five subjects (21.7%) had been taking were hypertension and hyperlipidemia medicine.

**Table 1:** Baseline characteristics of enrolled subjects.

Age (years)	54.17 ± 5.29
Body mass index (kg/m^2^)	24.28 ± 3.71
Duration of pain (years)	2.65 ± 2.79
**Current medication**	
No	18 (78.3%)
Yes	5 (21.7%)
Hypertension	4
Hyperlipidemia	2

Data are presented as mean ± standard deviation or %.

### 3.2. Primary outcomes

**Figure 3:**
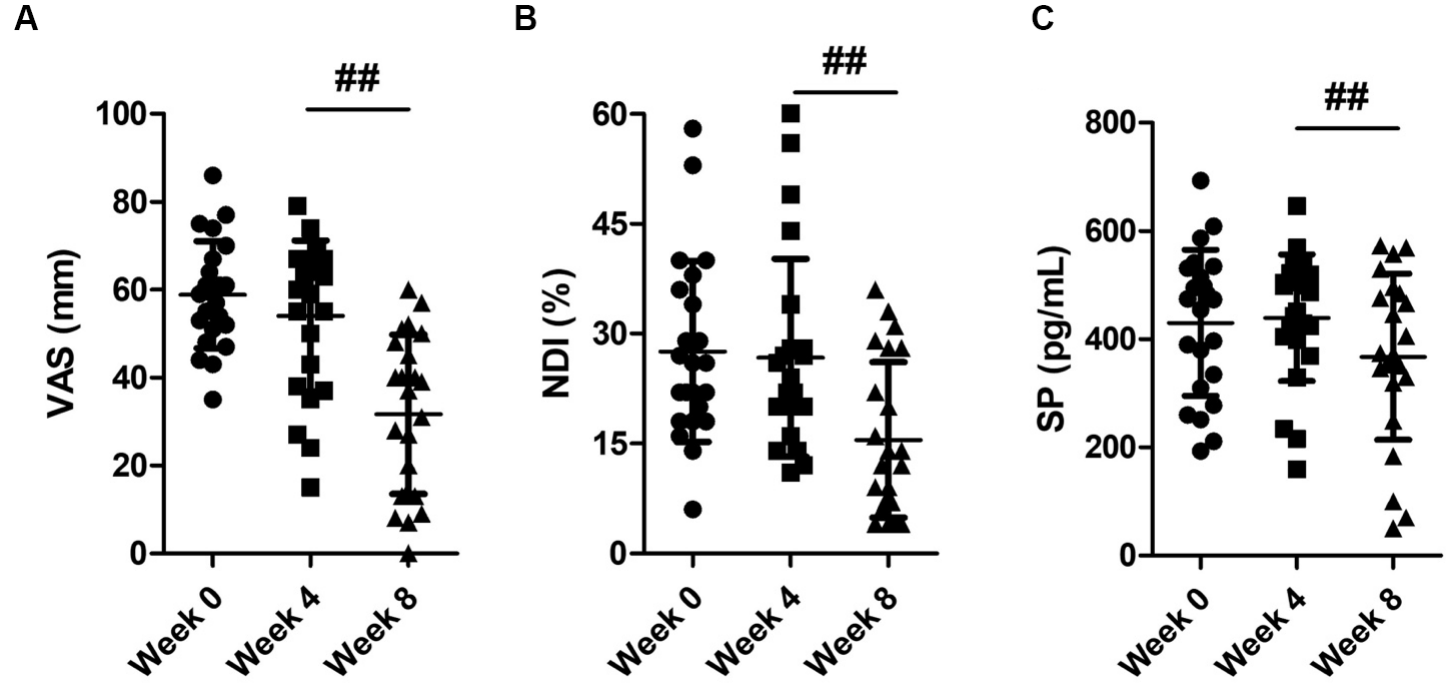
Changes in **(A)** visual analogue scale (VAS), **(B)** neck disability index (NDI), and **(C)** substance P (SP) level. Data are presented as mean ± standard deviation. The significance of the within-subject factor (time) was tested by one-way ANOVA with repeated measures. ^##^*p* < 0.001: week 4 vs. week 8.

Pain intensity measured using VAS showed no significant differences between measurement values at baseline and week 4 (*p* = 0.102). There was also no significant difference between the baseline NDI score and the score after 4 weeks-waiting (*p* = 0.618). According to the test of within-subjects effect for VAS result, a significant improvement in pain was observed after 4 weeks-treatment compared to the value after 4 weeks-waiting (*p* < 0.001). In addition, there was also a significant difference in NDI score between the score measured after 4 weeks-waiting and after 4 weeks-treatment (*p* < 0.001) (Figures 3A,B).

### 3.3. Secondary outcome

The results of one-way ANOVA with repeated measures showed no significantly different changes in SP between baseline value and after the 4 weeks-waiting (*p* = 0.736). However, there was a significant change in SP value after 4 weeks-treatment compared to the value examined prior to the treatments (*p* < 0.05) (Figure 3C).

## 4. Discussion

We observed efficacy of acupuncture on chronic neck pain by assessing perceived pain and changes of SP level in serum. There was no significant difference in the comparison of VAS score at baseline vs. after 4 weeks-waiting. After 4 weeks-acupuncture intervention on the subjects with chronic neck pain, there was significant alleviation in pain intensity as VAS decreased from 53.96 mm to 31.65 mm. Subjects also reported that their overall disability level in daily life had diminished after treatments (15.46 ± 10.65) compared to before treatments (26.69 ± 13.45). For pain assessment, patient-reported outcome measures have been widely applied in clinical trials. By self-evaluating pain intensity, patients can subjectively detect their pain over time (24). Our primary outcome data obtained from VAS and NDI also shows how effectively acupuncture improved pain in patients with chronic neck pain.

A growing number of studies demonstrate reliable evidence of the effect of acupuncture on various diseases despite ongoing controversies over acupuncture treatment. Several studies examined the effect of acupuncture treatment on different types of pain. Acupuncture treatment significantly decreased VAS score in Whiplash patients compared to the no-treatment control group (25). In our study, the VAS and NDI were decreased by an average of −22.31 mm and −11.24%, respectively, after 4 weeks of acupuncture intervention. According to the results we obtain from two different self-evaluations, the pain-relief effect of acupuncture on acute pain is also evident in chronic neck pain.

In previous pain-related research, a multitude of biomarkers have been studied to understand complex neurobiological mechanisms of pain. SP is an undecapeptide belonging to the tachykinin peptide family. It is widely distributed in the central and peripheral nervous systems and released upon nociceptive stimulation. SP is involved in numerous neuronal signaling pathways and plays a pivotal role as a neurotransmitter and a neuromodulator (26, 27). However, the effect of acupuncture on SP has not been well understood in patients with chronic neck pain. Inflammatory biomarkers often reflect the degree of inflammation. We explored the change in SP level to objectively measure pain and make a reliable connection between pain modulation and acupuncture treatment for chronic neck pain. The painful perception can occur by various factor, which means it could stimulate different neural pathways. Interestingly, in this study, SP levels significantly decreased after acupuncture intervention in subjects with chronic neck pain, whereas there was no significant difference in SP levels between baseline and week 4. Based on both primary and secondary outcomes, a correlation between the effect of acupuncture and changes in SP levels can be suggested. However, there were limited subjects and healthy comparison group to further suggest more concrete underlying mechanism. We still need to further investigate how acupuncture played a regulatory role in central sensitization which led to SP level changes and also identify whether SP level is directly or indirectly involved in potential therapeutic mechanisms of acupuncture such as segmental inhibition, release of endogenous opioid, and adrenergic and 5 HT pathway.

This study assessed the impact of acupuncture treatment chronic neck pain in a single group over time. It allowed us to minimize subjects’ variation and to compare pain modulation. Although more studies with larger sample sizes and the inclusion of a healthy control group are expected to provide more reliable evidence of the effect of acupuncture on pain regulation in general, our findings suggested the beneficial effect of acupuncture on chronic neck pain and potential pain relief mechanism.

## 5. Conclusion

The present study suggests that acupuncture alleviates chronic neck pain by managing pain intensity and improving neck pain-induced disabilities in daily life. However, it is still necessary to further investigate the biological mechanism underlying the role of acupuncture in pain relief based on the secondary outcome of this study.

## Data availability statement

The original contributions presented in the study are included in the article/supplementary material, further inquiries can be directed to the corresponding author.

## Ethics statement

The studies involving humans were approved by Institutional Review Board of Kyonggi University (KGU-20171222-HR-026). The studies were conducted in accordance with the local legislation and institutional requirements. The participants provided their written informed consent to participate in this study.

## Author contributions

JK: Investigation, Writing – original draft. S-NK: Conceptualization, Supervision, Writing – review & editing.
